# A bilberry drink with fermented oatmeal decreases postprandial insulin demand in young healthy adults

**DOI:** 10.1186/1475-2891-10-57

**Published:** 2011-05-21

**Authors:** Yvonne E Granfeldt, Inger ME Björck

**Affiliations:** 1 Department of Food Technology, Engineering and Nutrition, Lund University P.O. Box 124, SE-221 00 Lund, Sweden

**Keywords:** glucose response, insulin response, GI, blueberry, bilberry, rosehip

## Abstract

**Background:**

in traditional medicine, blueberries have been used to facilitate blood glucose regulation in type 2 diabetes. Recent studies in diabetic mice have indicated facilitated glycaemic regulation following dietary supplementation with extracts from European blueberries, also called bilberries, (*Vaccinium myrtillus)*. The purpose of the present study was to investigate the impact of fermented oat meal drinks containing bilberries or rosehip (*Rosa canina*) on glycaemic and insulinaemic responses.

**Methods:**

glycaemic and insulinaemic responses in young healthy adults were measured in two series. In series 1, two drinks based on oat meal (5%), fermented using *Lactobacillus plantarum *299v, and added with fruit (10%); bilberries (BFOMD) or rose hip (RFOMD) respectively, were studied. In series 2, BFOMD was repeated, additionally, a drink enriched with bilberries (47%) was tested (BBFOMD). As control a fermented oat meal drink (FOMD) was served.

**Results:**

in series 1 the bilberry- and rosehip drinks, gave high glucose responses similar to that after the reference bread. However, the insulin index (II) after the BFOMD was significantly lower (II = 65) (P < 0.05). In series 2 a favourably low insulin demand to BFOMD was confirmed. FOMD gave high glucose response (GI = 95) but, significantly lower insulin response (II = 76). BBFOMD gave remarkably low insulin response II = 49, and tended to lower glycaemia (GI = 79) (P = 0.0684).

**Conclusion:**

a fermented oat meal drink added with bilberries induced a lower insulin response than expected from the glycaemic response. The mechanism for the lowered acute insulin demand is still unclear, but may be related to some bio-active component present in the bilberries, or to the fermented oat meal base.

## Background

One important nutritional characteristic of carbohydrate foods concerns their impact on glycaemic regulation and insulin demand. Whereas the glycaemic response to starchy foods are influenced mainly by the rate of starch digestion and absorption, the gastric emptying rate and/or the motility in the small intestine [1], that of fruits may also be influenced by other characteristics. Consequently, the carbohydrate composition; starch, glucose, fructose and sucrose [2,3], the degree of ripeness, affecting the distribution of starch to low molecular weight carbohydrates, and the food structure [4] play a role. Additionally, the type and amount of organic acids present in berries might affect glycaemic regulation, in accordance with the benefits seen with organic acids produced upon sour-dough fermentation [5,6]. The glucose and insulin responses to carbohydrate foods have been extensively tested most of them being rich in starch rather than sugars [7]. The glycaemic and insulin responses to sugars are particularly relevant in juices rather than in intact vegetable or fruits, as drinks and juices may allow consumption of higher amounts of carbohydrates, thus having a greater impact on glycaemia. A major challenge of nutrition science is the combat of diet related disorders, in particular, diseases connected to the insulin resistance syndrome. Quality parameters of importance in this connection are the postprandial glucose and insulin responses, where food characterised by a low glycaemic index (GI) or glycaemic load (GL) have been found to induce benefits on several risk makers for this syndrome as judged from interventions in healthy and type 2 diabetic-subjects [8]. In fact, oscillatory hyperglycaemic episodes are considered to trigger production of inflammatory markers and oxidative stress, events that are increasingly being associated with endothelial damage, and risk of cardio-vascular disease [9]

Several members of the *Vaccinium *genus, including *Vaccinium myrtillus*, bilberry (European blueberry), closely related to blueberries, *Vaccinium angustifolium*, are considered to possess anti-diabetic activity, and are used in traditional medicine for the treatments of diabetic symptoms [10]. However, the majority of human and animal studies on blueberries and bilberries have focused on the anti-oxidative properties [11-14] as evaluated based on serum antioxidant status, and not on the potential effects on glycaemic control. Some in vitro results are available, though, showing potential anti-diabetic capacity of blueberries caused by the presence of specific bioactive components displaying insulin-like properties [15]. Further, recent studies in diabetic mice have shown decreased blood glucose with bilberry extract [16] and with fermented blueberry juice [17].

Although some studies have investigated the glycaemic response after mixed berries [18] and certain fruits [2,7,19,20], human data on glycaemic and insulinemic response to blueberries, bilberries or products made from these berries, are to our knowledge not available. The present study was performed to determine the glycaemic and insulinemic responses in healthy humans after single meal intakes of fermented oat meal drinks containing different amounts of bilberries (0, 10 or 47%) or rosehip (10%).

## Methods

### Experimental design

The study was divided in two series, series 1 with two fermented oatmeal drinks added with bilberry and rosehip, respectively, and series 2 with a fermented oatmeal reference drink without fruit, and with 2 oat meal drinks with bilberry added in different amounts. The effect of carbohydrate equivalent servings of these drinks on blood glucose and insulin responses was studied at breakfast in healthy young subjects. White wheat bread was used as a reference in both series allowing for calculation of glycaemic and insulinemic indices.

### Series 1

The two test products were; 1**) **a bilberry drink based on bilberry (10%), and, oatmeal (5%), fermented with *Lactobacillus plantarum *299v (BFOMD), and 2) a rose hip drink based on rose-hip (10%), and oatmeal (5%), fermented with *Lactobacillus plantarum *299v (RFOMD). The drinks were provided by (Skånemejerier, Malmö, Sweden) (ProViva^®^). As reference, a white wheat bread was baked under standardised conditions [21]. The test meals were standardised to contain 30 g available carbohydrates corresponding to bilberry drink (302 g), rosehip drink (300 g) and reference bread (70,3 g) (Table 1). The content of fluid in the two drinks was compensated for by providing 300 g of water with the reference bread meal.

**Table 1:** Composition of available carbohydrate in test meals and reference meal (series 1 and 2) (g wet weight)

Product	Glucose^1^	Fructose^1^	Sucrose^1^	Available starch^2^	Total available carbohydrates
**Series 1**					
Reference bread 70.2 g				30.0	30.0
BFOMD 302 g	2.7	2.9	20.5	3.7	29.8
RFOMD 300 g	0.9	0.8	26.9	1.35	29.9
**Series 2**					
Reference bread 70.0 g				30.0	30.0
FOMD 270.3 g	1.9	2.2	21.1	1.1	26.3
BFOMD 270.3 g	1.9	2.2	21.1	4.9	30.1
BBFOMD 307,7 g^3^	5.7	6.9	14.1	3.2	29.9

^1^Analysis with HPAEC (high pressure anion exchange chromatography)

^2^Available starch analysed according to [43]

^3^BFOMD contributed to two third of the available carbohydrates (180.2 g) and homogenised bilberries with one third (127.5 g)

#### Subjects

Nine healthy, non-smoking volunteers, 7 women and 2 men, took part in the study. Their average age was 32.7 ± 9.9 years (mean ± SD) and their mean body mass index 23.0 ± 2.4 kg/m^2 ^(mean ± SD). The night before every test breakfast, the subjects were requested to eat a standardised late evening meal, based on 2-3 slices of white wheat bread. After 10 pm, the subjects were allowed to drink only water. The reference- and test breakfast meals were served randomised after an overnight fasting. The tests were performed approximately one week apart and commenced at the same time in the morning. All meals were consumed steadily and completed within 12-14 min. Tea, coffee or water (150 ml) was served after each meal. The test subjects were allowed to choose between water, tea or coffee, and maintained the same drink through-out the study.

### Series 2

The test products in series 2 were 1) a bilberry drink based on bilberry (10%), and, oatmeal (5%), fermented with *Lactobacillus plantarum *299v (BFOMD) (ProViva^®^) 2) a fermented oatmeal drink (5%) (FOMD). The fermented oatmeal drink was supplemented with glucose (1,9 g/serving), fructose (2.2 g/serving) and sucrose (21.1 g/serving) to mimic the sugar composition in the bilberry drink, and 3) a bilberry drink BBFOMD, BFOMD added with frozen, thawed and homogenised bilberries. In the BBFOMD, the BFOMD contributed with two-thirds of the available carbohydrates, and homogenised bilberries with one-third. The test meals were standardized to contain 30 g available carbohydrates. Thus, the volunteers were served; 1) BFOMD (270.3 g), 2) FOMD (270.3 g), 3) BBFOMD (307,7 g), homogenised bilberries (127.5 g) added to BFOMD (180.2 g) and 4) 70.0 g reference bread (Table 1). The content of fluid in the two drinks was compensated for with 300 g of water being served with the reference bread. Tee, coffee or water (150 ml) was served after each meal. The test subjects were allowed to choose between these drinks and retained the same drink through-out the study.

#### Subjects

Eleven healthy, non-smoking volunteers, 7 women and 4 men, took part in the study. Their average age was 26.2 ± 4.6 years (mean ± SD) and their mean body mass index 23.5 ± 2.9 kg/m^2 ^(mean ± SD). The night before each test breakfast, the subjects were requested to eat a standardised late evening meal, based on 2-3 slices of white wheat bread. After 10 pm, the subjects were allowed to drink only water. The tests were performed approximately one week apart and commenced at the same time in the morning. All meals were consumed steadily and completed within 12-14 min.

### Sampling and analysis

A fasting blood sample was taken before the meal was served. After the breakfast, blood samples were taken at 15, 30, 45, 70, 95 and 120 min for analysis of glucose, and at 15, 30, 45, 95 and 120 min for analysis of insulin. Capillary blood was used.

Blood glucose concentrations were determined with a glucose oxidase peroxidase reagent [21] and serum insulin concentrations with an enzyme immunoassay kit (Mercodia Insulin Elisa; Mercodia AB, Uppsala, Sweden).

The Ethics Committee of the Faculty of Medicine at Lund University approved the study.

### Statistical analysis

The incremental areas under the curves were determined for blood glucose and serum insulin (GraphPad Prism version 4.03; GraphPad Software, San Diego, CA, USA). GI and II were calculated from the area under the glucose/insulin response (0-120 min) after consumption of 50 g of carbohydrates from a test food divided by the area under curve after consumption of 50 g of carbohydrates from white wheat bread (reference) and with each subject being their own reference. All areas below the baseline were excluded from the calculations. The relationship between the insulin and glucose response (II/GI) was used to predict the insulin demand for the test product. Values are presented as mean ± SEM. All statistical calculations were performed in MINITAB Statistical Software (release 13 for Windows; Minitab Inc., State College, PA). Significances were evaluated with the general linear model (analysis of variance) followed by Tukey's multiple comparisons test. Values of *P *< 0.05 were considered significant.

## Results

### Series 1

**Figure 1:**
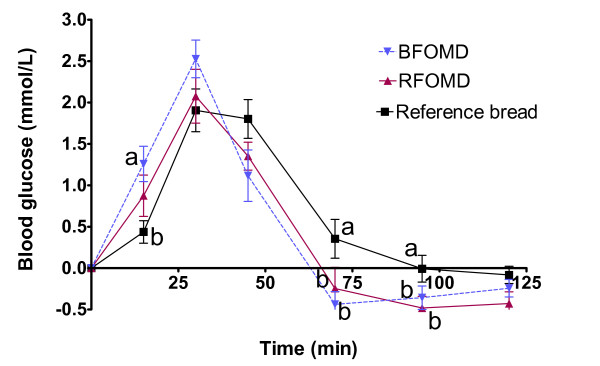
**(series 1) Mean incremental blood glucose responses in healthy subjects following ingestion of breakfast meals**. Mean values ± SEM, n = 11. Mean values with different letters at each time are significantly different (ANOVA followed by Tukey's test), P < 0.05.

The blood glucose responses after the drinks BFOMD and RFOMD, and the white reference bread are shown in Figure 1. At 15 min, the glucose response after the BFOMD was significantly higher than after the white wheat bread (P < 0.05). A similar tendency was observed at 30 min, but did not reach significance (P = 0.0595). The glucose response after both fruit drinks decreased more rapidly than after the reference bread. At 45 min there was a tendency to lower blood glucose response after the BFOMD compared with the reference bread (P = 0.0526), and at 70 and 95 min the blood glucose responses after both fruit drinks were lower than with the reference bread (P < 0.05).

The incremental areas under the postprandial glucose curves (0-120 min) and GI for the two fruit drinks were not significantly different from that with the reference bread (Table 2).

**Table 2:** Series 1.Glycaemic and insulinaemic data following breakfast meals with BFOMD, RFOMD and white wheat bread reference.

	Reference bread	BFOMD	RFOMD
Blood glucose:			
Fasting value (mmol/L)	4.5 ± 0.1^a^	4.4 ± 0.1^a^	4.5 ± 0.1^a^
Incremental area under curve (0-	88.1 ± 11.6^a^	78.6 ± 9.4^a^	73.4 ± 8.8^a^
120 min) (mmol min/L) GI (0-120 min) (%)	100^a^	95 ± 10^a^	87 ± 8^a^
			
Serum insulin:			
Fasting value (pmol/L)	65 ± 7^a^	81 ± 10^a^	80 ± 10^a^
Incremental area under curve (0-	14.7 ± 2.4^a^	9.4 ± 2.1^b^	10,0 ± 1.6^ab^*
120 min) (nmol min/L) II (0-120 min) (%)	100^a^	65 ± 6^b^	79 ± 16^ab^*
II/GI	1	0.68	0.9

Mean values ± SEM, n = 9. Mean values with different letters in each row are significantly different (ANOVA followed by Tukey's test), P < 0.05,

* comparing reference bread with RFOMD P = 0.0673

**Figure 2:**
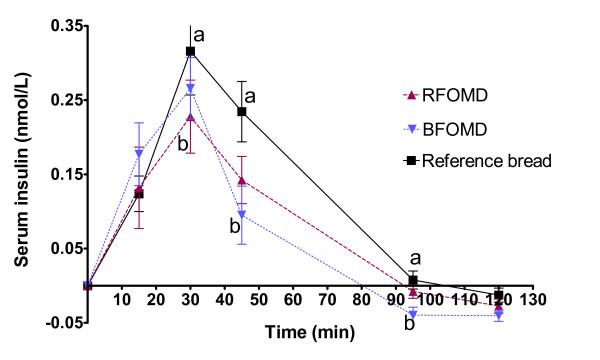
**(series 1) Mean incremental serum insulin responses in healthy subjects following ingestion of breakfast meals**. Mean values ± SEM, n = 11. Mean values with different letters at each time are significantly different (ANOVA followed by Tukey's test), P < 0.05.

The insulin responses are shown in Figure 2. At 30 min, the postprandial insulin response after the RFOMD was significantly lower than after the reference bread (P < 0.05), and at 45 and 95 min the insulin responses after the bilberry drink (BFOMD) were significantly lower than after the reference bread (P < 0.05). The incremental area under the postprandial insulin curve (0-120 min) after the BFOMD drink was significantly smaller than the corresponding area after the reference bread (P < 0.05). The corresponding area after the RFOMD drink also tended to be smaller than after the reference bread, but the difference did not reach significance (P = 0.0673) (Table 2).

### Series 2

**Figure 3:**
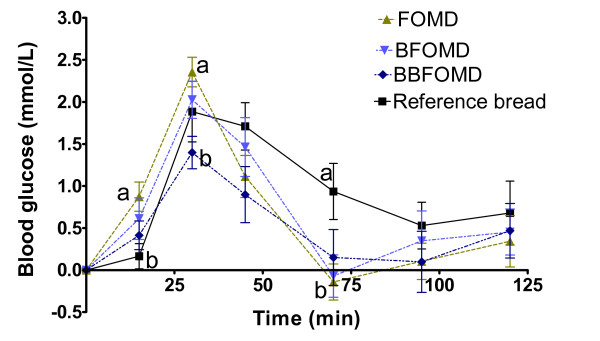
**(series 2) Mean incremental blood glucose responses in healthy subjects following ingestion of breakfast meals**. Mean values ± SEM, n = 11. Mean values with different letters at each time are significantly different (ANOVA followed by Tukey's test), P < 0.05.

The post prandial blood glucose responses are shown in Figure 3. At 30 min the glucose response after the FOMD was significantly higher than that after the BBFOMD (P < 0.05). Further, the FOMD gave a higher response than the reference bread at 15 min and a lower at 70 min (P < 0.05). The blood glucose responses after the two drinks with bilberry (BFOMD and BBFOMD) were not at any time point significantly different from each other, nor from the fermented oat meal base (FOMD) (except for at 30 min mentioned above) or from the reference bread. However, the incremental glucose area in the early postprandial phase (0-45 min), was significantly smaller after the BBFOMD (34.7 mmol min/L) compared with the FOMD (56.8 mmol min/L) (Table 3). Comparing the glycaemic areas (0-120 min), there was a tendency to a smaller area after BBFOMD than that after the reference bread but the difference did not reach significance (P = 0.0684).

**Table 3:** Series 2: Glycaemic and insulinaemic data following breakfast meals with FOMD, BFOMD, BBFOMD and white wheat bread

Variables	White wheat reference bread	FOMD	BFOMD	BBFOMD
Blood glucose:				
Fasting value (mmol/L)	4.8 ± 0.1^a^	4.8 ± 0.2^a^	4.9 ± 0.1^a^	5.1 ± 0.2^a^
Peak value at 30 min (mmol/L)	1.9 ± 0.4^ab^	2.4 ± 0.2^a^	2.2 ± 0.2^ab^	1.4 ± 0.2^b^
Incremental area under curve (0-45 min) (mmol min/L)	44.5 ± 6.9^ab^	56.8 ± 3.9^a^	51.5 ± 5.5^ab^	34.7 ± 3.4^b^
Incremental area under curve (0-120 min) (mmol min/L)	114.9 ± 15.1^a^	87.3 ± 8.5^a^	96.5 ± 14.3^a^	75.6 ± 13.7^a^*
GI (0-120 min) (%)	100^a^	95 ± 19^a^	94 ± 16^a^	79 ± 17^a^*
				
Serum insulin:				
Fasting value (pmol/L)	58.2 ± 5.5^a^	61.3 ± 7.9^a^	62.4 ± 8.9^a^	66.7 ± 9.3^a^
Incremental area under curve (0-120 min) (nmol min/L)	13.8 ± 2.5^a^	9.5 ± 1.5^b^	7.7 ± 1.2^b^	5.9 ± 1.0^b^
II (0-120 min) (%)	100^a^	76 ± 7^b^	63 ± 8^b^	49 ± 6^b^
II/GI	1	0.80	0.67	0.62

Mean values ± SEM, n = 11. Mean values with different letters in each row are significantly different (ANOVA followed by Tukey's test), P < 0.05

* P = 0.0684 comparing reference bread with BBFOMD.

**Figure 4:**
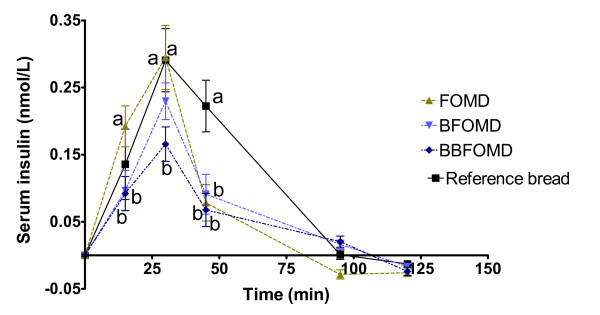
**(series 2) Mean incremental serum insulin responses in healthy subjects following ingestion of breakfast meals**. Mean values ± SEM, n = 11. Mean values with different letters at each time are significantly different (ANOVA followed by Tukey's test), P < 0.05.

Serum insulin responses are shown in Figure 4. The postprandial insulin responses after 15 min were significantly higher after the FOMD than after the two drinks with bilberries (BFOMD and BBFOMD) (P < 0.05). At 30 min the serum insulin response after the reference bread and the FOMD were significantly higher than that after the BBFOMD. At 45 min, all drinks, including the FOMD, BFOMD and BBFOMD, gave lower insulin responses than the reference bread. Also, the area under the insulin curves and the II-values were significantly smaller after FOMD and the bilberry drinks (BFOMD and BBFOMD) compared to the reference bread (P < 0.05, Table 3).

## Discussion

The two fruit drinks in series 1, BFOMD and RFOMD, gave a postprandial blood glucose response similar to that after an equivalent amount of carbohydrate from white bread. The GI was thus 97 and 89 for BFOMD and RFOMD, respectively. However, the high blood glucose responses were not accompanied by corresponding high insulin responses. Consequently, a tendency to a lower insulin response was present after both fruit drinks compared to that after white bread, even though only the area under the insulin curve after the BFOMD (0-120 min), was significantly smaller than after white reference bread. The II was determined to 65 (P < 0.05) and 79 (P = 0.0673) for BFOMD and RFOMD, respectively.

When calculating GI's, according to the content of digestible carbohydrates and their GI values (table 4), the BFOMD received a GI of 95 and the RFOMD a GI of 97. Accordingly, the determined GI values in series 1 (97 and 89 for BFOMD and RFOMD respectively) are in good agreement with those calculated. Previously, also Gannon *et al *[3] found that glucose response to fruits (oranges and apples) can be predicted from the constituent carbohydrates present, whereas the insulin response cannot. However, in contrast to our finding with low insulin responses Gannon et al found higher observed insulin responses than predicted from glycaemia in the case of orange- and apple juice.

**Table 4:** Available carbohydrate composition, and calculated GI (Series 1 and 2)

Product	Glucose	Fructose	Sucrose	Available starch	Calculated GI^1^
**Series 1**					
BFOMD					
Proportion of total amount of digestible carbohydrates (%)	9.1	9.9	68.6	12.5	
Contribution to GI	12.8	2.7	66.5	12.5	95
					
RFOMD					
Proportion of total amount of digestible carbohydrates (%)	3.0	2.7	89.8	4.5	
Contribution to GI	4.2	0.8	87.1	4.5	97
					
**Series 2**					
FOMD					
Proportion of total amount of digestible carbohydrates (%)	7.2	8.4	80.2	4.2	
Contribution to GI	10.2	2.3	77.8	4.2	95
					
BFOMD					
Proportion of total amount of digestible carbohydrates (%)	6.3	7.3	70.1	16.3	
Contribution to GI	8.9	2.0	68.0	16.3	95
					
BBFOMD					
Proportion of total amount of digestible carbohydrates (%)	19.1	23.1	47.2	10.7	
Contribution to GI	26.9	6.2	45.8	10.7	90

^1^Glucose GI = 141, Fructose GI = 27, Sucrose GI = 97, Starch GI = 100 [7]

In series 2, the GI and II for a BFOMD, matching that in series 1, were determined to 92 and 64, respectively compared with GI = 97 and II = 65 in series 1. Thus, the favourable effect of a fermented oat meal drink with bilberry on insulin demand in series 1 could be repeated. The FOMD gave a high GI (GI = 95) whereas that of the BBFOMD was lower (GI = 79). Insulin indices (II) for the FOMD and the BBFOMD were; II = 76 and 49, respectively. The II for the BBFOMD was remarkably low, consequently when using white bread as a reference, beverages like soft drinks (II = 97-118) [19], and other fruit/berry based drinks like orange-(II = 78) [19] or apple juice (II = 54, (estimated from insulin areas) [3] BBFOMD has a low II. Apple juice and BBFOMD resembling, that after e.g. pasta products (II = 35-53) [22].

The content of carbohydrates in the different products in the present study was similar, the amount of sugars being high (approximately 90%) and that of starch low. When calculating GI values according to the content of digestible carbohydrates and their GI values (table 4), the three test drinks in series 2, FOMD, BFOMD and BBFOMD received; 95, 95 and 90 respectively, to be compared with the determined GI values; 95, 94 and 79. The observed GI value for the BBFOMD was thus lower than expected; but for the other drinks the calculated and determined GI values were in good agreement. The decreased acute glycaemic response with a larger amount of bilberries (145.5 g) is noteworthy, and a hypothesis may be that the bilberries cause an increased uptake of glucose into the peripheral cells. Thus, Martineau *et al *[15] found insulin-like properties of ethanol extracts from Canadian low bush blueberries (*Vaccinium angustifolium)*, another member of the *Vaccinium *genus. The insulin-like properties were evident from enhancement of glucose uptake in differentiated muscle cells and adipocytes using an in vitro assay. Bilberry extract was also shown to reduce blood glucose level and enhance insulin sensitivity in type 2 diabetic mice via activation of AMPK (AMP-activated protein kinase), an enzyme central in the regulation of fuel preference, in adipose tissue, muscle- and liver cells [16]. These results support our finding of a decreased acute glycaemic response with bilberry and show that bilberry (*Vaccinium myritillus*) may contain active molecules with potential anti-diabetic properties. Such an effect, if present, is also coherent with the lowered insulin demand with bilberry drinks seen in both series 1 and 2. The existence of a dose-response relation between intake of bilberries and the corresponding glucose/insulin responses is currently in progress.

In this study we show an inconsistency between glycaemic and insulinaemic responses, especially in the case of the products containing bilberries. Consequently a high glucose response was accompanied by a comparatively low insulin response. The insulin demand, if expressed as a relationship between insulinaemia and glycaemia (II/GI), was low, 0.62-0.68. To our knowledge, this apparent discrepancy with a low insulin response in parallel to a high glucose response has only been reported previously for fermented whole-grain oat [23], certain rye products [24] and for cinnamon added to a rise pudding [25]. Earlier studies generally have shown a good correlation between glucose and insulin responses. Thus, studies with cereals [26], and certain fruits like mango, melon, pineapple, kiwi, apple and black grapes [19,27] indicate good agreement between GI and II. In contrast studies with oranges and apples [28] as well as juice from these fruits [3,29] were reported to display unexpectedly high insulin responses. Similarly, a discrepancy between GI and II, with unexpectedly high insulin responses has been shown, for milk and milk products [30-32]. Consequently despite extremely low GI (GI = 15-30) for regular and fermented milk, the II values were high (II = 90-98) [31], probably due to an insulinotrophic effect of whey protein [33]. One cause for the beneficial metabolic effects of a low glycaemic diet is probably a lower insulin response [34,35], and increased insulin sensitivity [36]. The present findings of low insulin demand following bilberry drinks might thus indicate advantageous metabolic properties.

The fermented oat meal base of the drinks, with sugars added to mimic the BFOMD was included as a reference drink (FOMD). A comparison between the FOMD and the BFOMD, gave no significant differences in glucose or insulin responses. Also, the insulin response to the FOMD (II = 76) was significantly lower than for white bread (II = 100). In a previous study with oats (oat porridge and oat flakes) no differences in glucose- or insulin responses were seen compared with a white bread [37]. This indicates that the fermentation process per se may decrease insulin response to oats. It is also supported by a study with fermented whole grain oat showing a lower insulin response than would be expected from the glucose values [23]. However, the magnitude of insulin decrease in the post prandial phase was more pronounced when more bilberries were included in the meal. Of interest in this respect are results from an in vitro study evaluating the effect of fermented blueberry juice (intrinsic micro flora of blueberries) on glucose uptake and transport into muscle cells and adipocytes. Treatment of cells with fermented juice potentiated glucose uptake by 48% in C2C12 (mouse myoblast cell line) myotubes, and by 142% in 3T3-L1 adipocytes, whereas non fermented juice had no effect on glucose transport [38]. Treatment of cells with fermented blueberry juice was shown to activate AMPK. The authors thus suggest an insulin-independent pathway to be the mechanism for an increased glucose uptake [38]. A follow-up study in obese and diabetic mice showed that fermented blueberry juice decreased hyperglycaemia, in part due to increased adiponectin levels. However, no positive effects were seen on insulin levels [17]. In the presently reported study, we saw a decreased early glucose response after BBFOMD. However, insulin responses were significantly lower, or close to being significantly lower for the drink with rose hip (RFOMD) (P = 0.0673), and for all fermented test drinks whether containing bilberries or not. Whether the low insulin demand (II/GI) shown in the present study could be an effect of fermentation of bilberries is currently under investigation.

All tested drinks had a low pH, or about 3 for the bilberry containing drinks, and about 4 for the fermented oat meal drink (FOMD). A low pH may lower post prandial glycaemia and hormonal responses due to e.g. a lowering of the rate of gastric emptying [39]. However, such a mechanism should preferably affect both blood glucose and insulinaemia to a similar extent.

Berries like bilberries and blueberries are known to be a rich source of bioactive molecules like phenolic and antocyanin contents [40] and phenolic acids [41]. Besides that they both are powerful antioxidants they may also exert effects on other important biological systems as glucose- and insulin response. Bilberries mixed with blackcurrants, cranberries and strawberries (150 g), other berries rich in antocyanins, have recently been shown to decrease the peak glucose increment of 35 g sucrose in healthy subjects [18]. Also in type-2 diabetic mice, anthocyanins in bilberry have been suggested to reduce blood glucose levels and enhance insulin sensitivity [16]. Water soluble polyphenols isolated from cinnamon has been shown to have strong insulin-enhancing activity on cultured fat cells in vitro, [42]. Also, when tested in healthy subjects, cinnamon (3 g) added to a rice pudding (300 g) was shown to reduce post prandial serum insulin, but not glucose, levels compared to a rice pudding without cinnamon [25]. It is impossible to draw any conclusions regarding the effects of the antocyanins and/or polyphenols present in the oat meal based fruit drinks (RFOMD, BFOMD, BBFOMD) in the present study. However, it cannot be excluded that such components might have contributed to the low insulin demand seen after the fermented oat meal drink added with bilberries.

To our knowledge no meal studies have been published showing impact of bilberries on glycaemic and insulinaemic responses in humans.

## Conclusions

In the present study in healthy volunteers, we found that fermented oatmeal drinks added with bilberries reduced insulin demand to a considerable extent, with the fermented oat meal blueberry drink enriched with bilberries also being capable of reducing glycaemia. The mechanism remains obscure, and provides an interesting area for further investigations.

## List of abbreviations

GI: glycaemic index; GL: glycaemic load; II: insulin index; BFOMD: bilberry drink based on bilberry (10%), and, oatmeal (5%), fermented with *Lactobacillus plantarum *299v; RFOMD: rose-hip drink based on rose-hip (10%), and, oatmeal (5%), fermented with *Lactobacillus plantarum *299v; BBFOMD: bilberry drink BFOMD added with homogenised bilberries (41%); FOMD: fermented oatmeal drink (5%) fermented with *Lactobacillus plantarum *299v; BMI: body mass index.

## Competing interests

The authors declare that they have no competing interests.

## Authors' contributions

YEG participated in the design, conducted research, analyzed data and statistical analyses, wrote the paper

I.MEB participated in the design and wrote the paper.

Both authors read and approved the final manuscript.
